# Acorn Consumption Improves the Immune Response of the Dung Beetle *Thorectes lusitanicus*

**DOI:** 10.1371/journal.pone.0069277

**Published:** 2013-07-16

**Authors:** José R. Verdú, José L. Casas, Vieyle Cortez, Belén Gallego, Jorge M. Lobo

**Affiliations:** 1 I.U.I. Centro Iberoamericano de la Biodiversidad, Universidad de Alicante, San Vicente del Raspeig, Alicante, Spain; 2 Red de Ecoetología, Instituto de Ecología, A.C., Carretera a Coatepec No. 351, El Haya, A. P. 63, Xalapa, Veracruz, México; 3 Museo Nacional de Ciencias Naturales-Consejo Superior de Investigaciones Científicas, Department de Biodiversidad y Biología Evolutiva, Madrid, Spain; CNRS, Université de Bourgogne, France

## Abstract

*Thorectes lusitanicus*, a typically coprophagous species is also actively attracted to oak acorns, consuming, burying them, and conferring ecophysiological and reproductive advantages to both the beetle and the tree. In this study, we explored the possible relation between diet shift and the health status of *T. lusitanicus* using a generalist entomopathogenic fungus (*Metarhizium anisopliae*) as a natural pathogen. To measure the health condition and immune response of beetles, we analysed the protein content in the haemolymph, prophenoloxidase (proPO) content, phenoloxidase (PO) activity and mortality of beetles with diets based on either acorns or cow dung. Protein content, proPO levels and PO levels in the haemolymph of *T. lusitanicus* were found to be dependent on the type of diet. Furthermore, the beetles fed with acorns developed a more effective proPO-PO system than the beetles fed with cow dung. Furthermore, a significant decrease in mortality was observed when infected individuals were submitted to an acorn-based diet. In addition to enhancing an understanding of the relevance of dietary change to the evolutionary biology of dung beetles, these results provide a more general understanding of the ecophysiological implications of differential dietary selection in the context of fitness.

## Introduction

Despite the important effects of diet shift on fitness and the methodological improvements in the rapid determination of health status, studies covering these topics are scarce in insects. Such studies are important in that knowledge about the influence of nutrition on health status is critical for the understanding of many topics in ecology [1], population dynamics [2], and immunology [3]. For example, in *Spodoptera* moth caterpillars, protein-rich diets enhanced immunity to a viral pathogen compared to individuals fed on carbohydrate-rich diets [4], and a similar pattern occurred for antibacterial activity [5]. In the migrating Mormon cricket *Anabrus simplex*, a protein-rich diet also enhanced immunity against wounds and pathogen invasion [6]. However, cross-relationships between diet and infection susceptibility are complex and may depend on the pathogen and the species infected [3]. Thus, although the shift to a protein-rich diet could become a widespread process of “self-medication” to enhance the immunity of insects [7], some immune traits may also be associated either with high carbohydrate consumption or an interaction between protein and carbohydrate consumption [8].

Insect immunity is routinely measured by estimating the activity of the enzyme phenoloxidase (PO) [9]. PO is an oxidoreductase and is involved in the oxidation of phenols to quinones and the polymerisation of quinones to melanin [10]. Quinones and melanin generated by PO are toxic to microorganisms [11], [12]. Although other traits can be examined to estimate the immune response, there are several reasons to select PO activity as a surrogate for immune defence: i) PO is a major component of the insect immune system, and its activity has profound fitness consequences for many taxa [13], [14], [15]; ii) PO activity can be heritable [16], [17]; and iii) PO activity correlates with resistance to parasites/pathogens [17], [18], [19], [20], [21], veterinary drugs [22], and cuticular melanisation after moulting [23]. PO exists in the haemolymph as an inactive intracellular proenzyme, pro-phenoloxidase (proPO), which can be activated by effectors, such as β-1,3-glucan, peptidoglycan or lipopolysaccharides [10], [12], [24], [25], [26]. Although knowledge of the synthesis of this enzyme, its polymorphisms, regulation, activating enzymes and inhibitors is still limited and controversial (for revision, see [10]), its relevance for evaluating insect immunity is clear.

*Thorectes lusitanicus* is a Mediterranean ecosystem species adapted to eat faeces of herbivores (dung-fiber consumers). This species is classified within the dung beetles as a ‘telephagic tunneller’ since it has the ability to ship dung from source to the nesting site. However, in oak forests of the southeastern Iberian Peninsula, *T. lusitanicus* is surprisingly also actively attracted to oak acorns (*Quercus suber* and *Q. canariensis*), consuming and burying them both under laboratory and field conditions [27]. Under laboratory conditions, this species prefers acorns in comparison to the dung of large herbivores, which has been described as the main food source for this and other taxonomically related dung beetle species [28]. From a nutritional point of view, this surprising dietary shift confers notable ecophysiological and reproductive advantages to *T. lusitanicus* [29]. The haemolymph composition of this beetle is greatly altered by its diet, which is particularly observed through changes in protein and fatty acid content. In particular, the haemolymph protein content of *T. lusitanicus* beetles feeding on acorns is 4.6 times higher than the beetles consuming cow dung [29].

As immune assays should be performed with relevant pathogens [21], we used a generalist entomopathogenic fungus (*Metarhizium anisopliae*) that is currently used as a biological insecticide [30] to explore the relation between diet and health status in *T. lusitanicus*. This entomopathogenic fungus has been currently observed in several scarabaeid beetles species [31], [32], [33] including *T. lusitanicus* (personal observation) what it is important to assure the pathogenic capacity of the conidia formulation. We examined temporal changes in the total protein contents, proPO, PO and variation in the mortality rates of inoculated individuals submitted to diets based on acorns or cow dung.

## Materials and Methods

### Insects

This work conforms to the Spanish legal requirements including those relating to conservation and welfare. Also, beetle collection was made with relevant permissions related to collection and field study in the Los Alcornocales Natural Park.

Individuals of *Thorectes lusitanicus* were captured from Los Alcornocales Natural Park in the Aljibe Mountains of southern Spain (36°31′54′′N, 5°34′29′′W) during November 2011. We used pitfall traps baited with cow dung to capture live beetles in four 48-h periods. The dung beetles were maintained in plastic containers (60×40×40 cm) at 20°C, and they were transferred to climate chambers at 20°C upon their arrival to the laboratory. Several requirements were considered to ensure a homogeneous physiological state of all specimens, thus allowing for the accurate comparison of physiological measures between the two diets [29]. We selected mature specimens (sex ratio of 1∶1) based on the cuticular deterioration of the anterior tibia in conjunction with the hardness of the pronotum and elytra cuticle [34], which allowed us to identify individuals of approximately the same age. To avoid the effect of diet and gut content in the field, all of the beetles were starved for a ten-day acclimation period prior to the captive feeding period.

### Types of Diet and Bioassay Design

To examine the immune response to pathogen inoculation, 10 individuals of *T. lusitanicus* (5 males and 5 females) were placed in one of eighteen plastic containers (30×20×15 cm) with sterile dry vermiculite. The specimens of nine containers were fed exclusively with acorns of *Quercus suber*, while the remaining specimens were fed exclusively with cow dung. The containers were placed in a climate chamber at 25°C with 65% relative humidity (RH) and a photoperiod of 15∶9 (light:dark).

### Entomopathogenic Fungus, Infection and Pathogen Resistance

To assess the influence of diet on the immune response and pathogen resistance, we exposed all individuals of *T. lusitanicus* to *Metarhizium anisopliae*. The fungus was isolated from a field-infected individual of *T. lusitanicus* and was plated onto potato dextrose agar (PDA, Sigma-Aldrich Co.) in Petri dishes (9 cm) at 28°C using an MIR-153 programmable heated and cooled incubator (SANYO Electric Co. Ltd, Japan) with an accuracy of 0.2°C. After isolation, *M. anisopliae* conidia were grown on PDA in 25 Petri dishes (9 cm) at 28°C. After five days, the conidia were well developed and collected from the Petri dishes by rasping into 50 ml of 0.1% sterile Tween 80 (Sigma-Aldrich Co.) using sterile scalpels. To obtain a spore formulation, the suspension was diluted to a final concentration of 1×10^6^ conidia per millilitre. The concentration of conidia was calculated using an improved Neubauer haemocytometer (Laboroptik Ltd, UK). Prior to fungus inoculation (t1 = 0), an initial sample of haemolymph was extracted as described below. In each plastic container, 20 ml of conidia suspension was uniformly sprayed in each container to ensure that all individuals were completely covered by the suspension. At four (t2) and eight days (t3) after inoculation, three containers from each treatment (diet) were randomly selected, and the haemolymph from all of the individuals was extracted. During the bioassay, the beetles were supplied with cow dung or acorns every four days to ensure that the food supply was not a limiting factor in the analysis.

### Haemolymph Collection

Haemolymph samples were obtained for each diet, sex and time interval (t1, t2 and t3) of conidia treatment. Haemolymph collection was performed by piercing the dorsal surface of the pronotum with a sterile pin and collecting the haemolymph with sterile capillary tubes. Each individual extraction was transferred to a 1.5-ml Eppendorf vial, and the haemolymph from five individuals was mixed to form a single sample per vial. A total of 36 haemolymph sampling units were available for statistical analyses corresponding to the pooled values of five male or female individuals (3 time periods×2 sexes×2 treatments×3 replicates). After the haemolymph extraction, the samples and the beetles were frozen and stored at –85°C in an ultrafreezer (SANYO Electric Co. Ltd, Japan).

### Total Protein Measurement

The haemolymph protein content was calculated using the Bradford method [35]. Briefly, the extracted haemolymph was centrifuged at 20,000×g for 5 minutes at 5°C to separate the particulate material and diluted 3∶100 or 1∶100 in water for cow dung- or acorn-fed beetles, respectively. Aliquots of 50 µl were then mixed with 1,500 µl of the Bradford reagent and left to stand at room temperature for 10 minutes. The absorbance was measured at 595 nm in a Shimadzu UV-6003 UV-VIS spectrophotometer (Shimadzu Scientific Instruments Ltd, Japan). The protein concentration was calculated from a calibration plot constructed under the same conditions using bovine serum albumin (BSA) as a standard.

### Assays of Phenoloxidase and Pro-phenoloxidase Activation

The measurements of PO activity were conducted as previously described [36] with slight modifications. Briefly, PO and proPO in the haemolymph were assayed spectrophotometrically by recording L-3,4-dihydroxyphenylalanine (L-DOPA, Sigma-Aldrich Co.) oxidation at 492 nm in a Shimadzu UV-1603 UV-VIS spectrophotometer equipped with a Shimadzu TTC-controller set at 25°C. Prior to measuring, aliquots of 20 or 10 µl of haemolymph from cow dung- and acorn-fed beetles, respectively, were diluted to 200 µl in 50 mM phosphate buffered saline (PBS, pH 7.4) and submitted to two cycles of freeze-thawing to disrupt the cells and release their content. The defrosted haemolymph was then centrifuged for 5 min at 20,000×g at 5°C and used immediately. To assay PO activity, 100 µl of the haemolymph solution was mixed with 100 µl of 20 mM L-DOPA, and the absorbance was registered at 492 nm every 0.1 sec for 30 minutes. The linear phase was typically reached between 3 and 25 minutes. PO activity is expressed as units per ml of haemolymph, where one unit is the amount of enzyme required to increase the absorbance by 0.001 units per minute.

ProPO is known to become activated to PO [37]. To examine this activation, 200 µl of haemolymph was incubated with 5 µl of Type IX-S Trypsin (Sigma-Aldrich, Co.) (1.67 mg/ml) for 5 min at 25°C. Then, 200 µl of 20 mM L-DOPA was added, and the total PO activity was measured as described above.

### Diet Effect on Mortality

**Figure 1:**
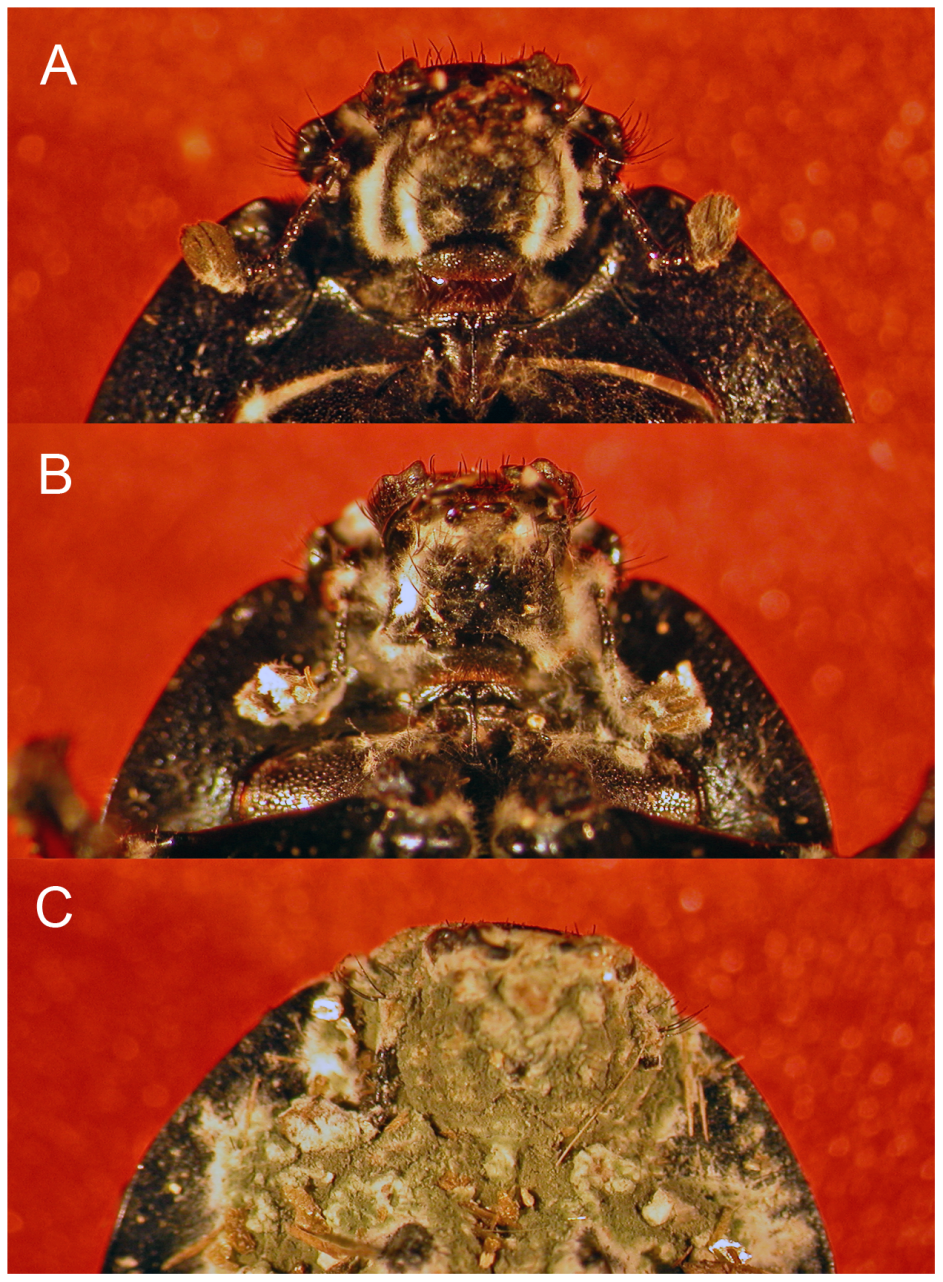
*Thorectes lusitanicus* at various stages of infection with *Metarhizium anisopliae*. A) First appearance of mycelia (in white) crossing the intersegmental membrane in the mouthparts; B) Advanced stage of infection showing mycelia crossing all the intersegmental membranes; and C) Individual showing the characteristic green conidia of *M. anisopliae*.

To study the effect of diet on mortality, 10 individuals of *T. lusitanicus* (5 males and 5 females) were placed into one of twenty-four sterile aluminium containers (30 cm in diameter) with sterile dry vermiculite. The specimens of twelve containers were fed exclusively with acorns of *Quercus suber*, while the remaining specimens were fed exclusively with cow dung. The containers were placed in a climate chamber at 25°C with 75% relative humidity (RH) and a photoperiod of 15∶9 (light:dark). As mentioned above for the PO and proPO assays, 20 ml of conidial suspension was sprayed uniformly in each plastic container to assure that all individuals were completely covered by the suspension. Mortality was checked three times every four days after the exposure to *M. anisopliae* conidia. For each time period (t1 = 4 days, t2 = 8 days and t3 = 12 days), the number of male and female *T. lusitanicus* that succumbed to *M. anisopliae* were counted. The beetles that succumbed showed the typical mycelia growth that traversed the intersegmental membranes (Fig. 1).

### Statistical Analyses

The data were analysed by a factorial ANOVA design using General Linear Models (GLM) after a log transformation of the dependent variables (protein, PO and proPO content) to normal distribution. Normality was examined using Shapiro-Wilk’s test (P>0.05 in all cases after logarithmic transformations). As in a factorial design both main effects and interactions are considered simultaneously, testing for differences in the response variable between treatment groups should be carried out re-running the model excluding interactions when they are nonsignificant [38]. The software STATISTICA v8.0 [39] was used for all statistical analyses.

## Results

### Effect of Diet and Infection on proPO Content and PO Activity

**Figure 2:**
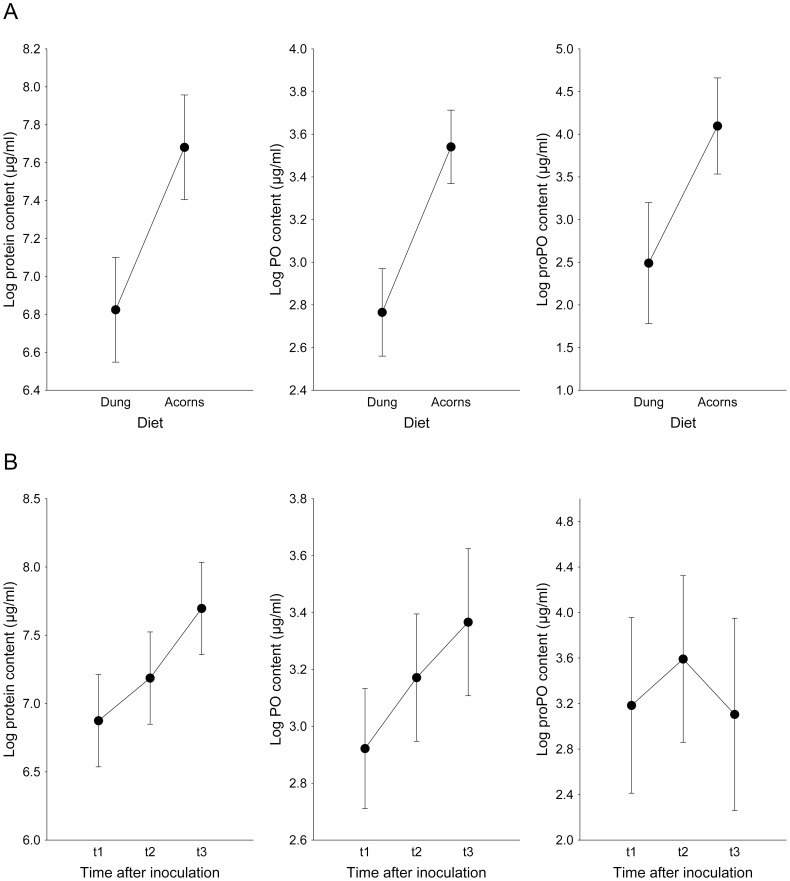
Total protein content (±95% confidence interval) and phenoloxidase (PO) and prophenoloxidase (proPO) concentrations in the haemolymph of *T. lusitanicus* individuals based on diet (A; dung or acorns), and the time after the inoculation of the pathogen *Metarhizium anisopliae* (B; t1 = 0 days, t2 = 4 days and t3 = 8 days after fungus inoculation).

The diet and time after inoculation were the only statistically significant factors for PO, whereas diet was the only significant factor for proPO (Table 1; Fig. 2). The phenoloxidase content was higher in the beetles feeding on acorns (mean: 37.2 µg/ml; minimum: 20.0 µg/ml; maximum: 75.0 µg/ml) than in those feeding on dung (mean: 16.3 µg/ml; minimum: 5.0 µg/ml; maximum: 22.5 µg/ml), a pattern repeated in the case of pro-phenoloxidase (mean: 99.8 µg/ml; minimum: 1.3 µg/ml; maximum: 264.7 µg/ml for the acorn diet, and mean: 19.9 µg/ml; minimum: 3.0 µg/ml; maximum: 79.1 µg/ml for the dung diet) (Fig. 2A). The pattern of PO observed over time was similar to that observed for protein content (Fig. 2B). For the acorn diet, mean PO activity were 26.7 U/min/ml (minimum: 20.0 U/min/ml; maximum: 40.0 U/min/ml), 38.3 U/min/ml (minimum: 25.0 U/min/ml; maximum: 75.0 U/min/ml) and 46.7 U/min/ml (minimum: 35.0 U/min/ml; maximum: 75.0 U/min/ml), for each t1, t2 and t3 time periods, respectively. For beetles feeding on dung, mean PO activity were 14.6 U/min/ml (minimum: 5.0 U/min/ml; maximum: 17.5 U/min/ml), 16.0 U/min/ml (minimum: 12.5 U/min/ml; maximum: 17.5 U/min/ml), and 19.4 U/min/ml (minimum: 17.5 U/min/ml; maximum: 22.5 U/min/ml), for t1, t2 and t3 time periods, respectively. However, the time after inoculation did not seem to affect the proPO content (Table 1; Fig. 2B). For the acorn diet, mean proPO content were 75.4 U/min/ml (minimum: 1.3 U/min/ml; maximum: 259.5 U/min/ml), 148.4 U/min/ml (minimum: 17.3 U/min/ml; maximum: 264.7 U/min/ml), and 75.6 U/min/ml (minimum: 36.0 U/min/ml; maximum: 160.0 U/min/ml), for t1, t2 and t3 time periods, respectively. For beetles feeding on dung, mean proPO content were 18.8 U/min/ml (minimum: 12.5 U/min/ml; maximum: 30.0 U/min/ml), 23.6 U/min/ml (minimum: 3.0 U/min/ml; maximum: 79.1 U/min/ml), and 16.3 U/min/ml (minimum: 4.0 U/min/ml; maximum: 28.7 U/min/ml), for t1, t2 and t3 time periods, respectively.

**Table 1:** Results of the factorial ANOVA design using General Linear Models between protein, phenoloxidase (PO) and pro-phenoloxidase (proPO) content in the haemolymph of *Thorectes lusitanicus* and the three considered factors: diet, time from exposure to *Metarhizium anisopliae* and sex of the beetles.

		Protein		PO		proPO	
	d.f.	*F*	P	*F*	P	*F*	P
Diet	1	20.51 (19.67)	0.0001 (0.0001)	36.21 (44.54)	<0.0001 (<0.0001)	13.79 (12.94)	0.001 (0.001)
Time	2	6.42 (6.16)	0.005 (0.005)	3.97 (5.73)	0.03 (0.008)	0.51	ns
Sex	1	0.01	Ns	0.04	ns	0.43	ns
Time*Diet	2	1.71	Ns	0.32	ns	1.27	ns
Time*Sex	2	1.72	Ns	0.28	ns	1.04	ns
Diet*Sex	1	0.09	Ns	2.04	ns	0.21	ns
Time*Diet*Sex	2	1.19	Ns	0.03	ns	1.93	ns

The results obtained by re-running the model excluding the interactions are showed in brackets.

### Relationship between Protein Content and PO and proPO Activity

Both the diet type and the time after fungus inoculation significantly affected the haemolymph protein content of *T. lusitanicus* (Table 1; Fig. 2). The beetles with an acorn-based diet showed higher total protein content in the haemolymph (mean: 2,692.1 µg/ml; minimum: 678.4 µg/ml; maximum: 6,381.5 µg/ml) than the beetles feeding on cow dung (mean: 1,053.7 µg/ml; minimum: 205.6 µg/ml; maximum: 1,986.4 µg/ml). For the acorn diet, mean PT content was 1,503.1 µg/ml (minimum: 683.0 µg/ml; maximum: 2,448.9 µg/ml), 2,082.1 µg/ml (minimum: 678.4 µg/ml; maximum: 4,351.3 µg/ml), and 4491.2 µg/ml (minimum: 2,607.2 µg/ml; maximum: 6,381.5 µg/ml), for t1, t2 and t3 time periods, respectively. For beetles feeding on dung, mean PT content was 794.9 µg/ml (minimum: 205.6 µg/ml; maximum: 1,126.2 µg/ml), 1173.2 µg/ml (minimum: 221.9 µg/ml; maximum: 1,759.3 µg/ml), and 1,193.0 µg/ml (minimum: 672.2 µg/ml; maximum: 1,986.4 µg/ml), for each t1, t2 and t3 time periods, respectively. If we considered only the factor time, a Scheffe post hoc test showed that the beetles have a significantly higher protein content eight days after inoculation (t3; mean: 2,842.1 µg/ml; minimum: 672.2 µg/ml; maximum: 6,381.5 µg/ml), than in the case of specimens prior to fungus inoculation (t1; mean: 1,149.0 µg/ml; minimum: 205.6 µg/ml; maximum: 2,449.0 µg/ml). However, the beetles four days post-inoculation (t2; mean: 1,627.7 µg/ml; minimum: 222.0 µg/ml; maximum: 4,351.3 µg/ml) do not have statistically significant differences in protein content regarding t1 or t3 specimens. The sex and the interaction terms were not statistically significant (Table 1).

**Figure 3:**
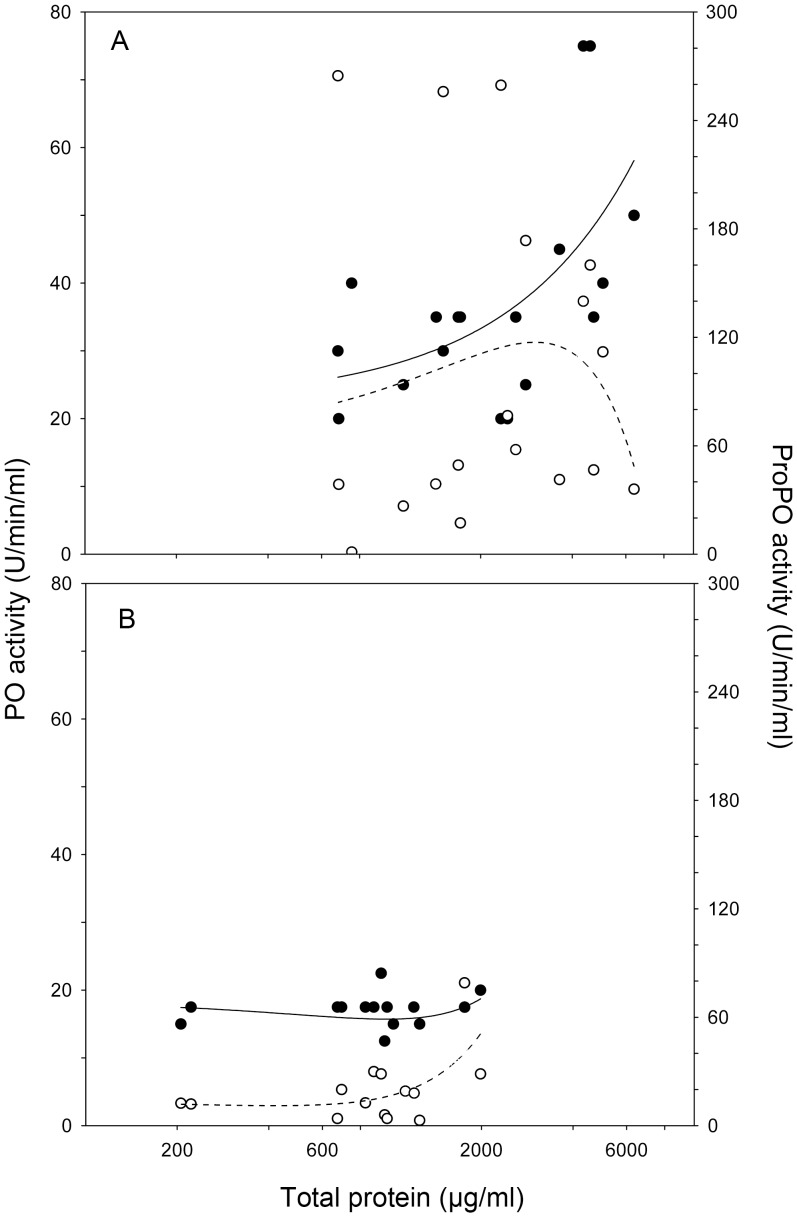
Relationships between the total protein content and phenoloxidase (PO) and prophenoloxidase (proPO) activities for *T. lusitanicus* beetles feeding on acorns (A) or cow dung (B). The filled and open circles represent PO and proPO measures, respectively. The least square regressions are fitted to show the possible patterns of both proPO and PO as a function of protein content.

In acorn-fed beetles, the total protein content was positively correlated with the phenoloxidase reaction rate (PO) in the haemolymph (Pearson correlation coefficient, *r* = 0.53; *P* = 0.02; n = 18) but not with pro-phenoloxidase (proPO) (*r* = 0.30; *P* = 0.22) (Fig. 3A). Conversely, neither PO nor proPO were significantly correlated with the total protein content in the haemolymph when the beetles were fed on dung (*r* = 0.22; *P* = 0.48 and *r* = 0.23; *P* = 0.46, respectively) (Fig. 3B).

### Effect of Diet on the Survival of Inoculated Insects

**Figure 4:**
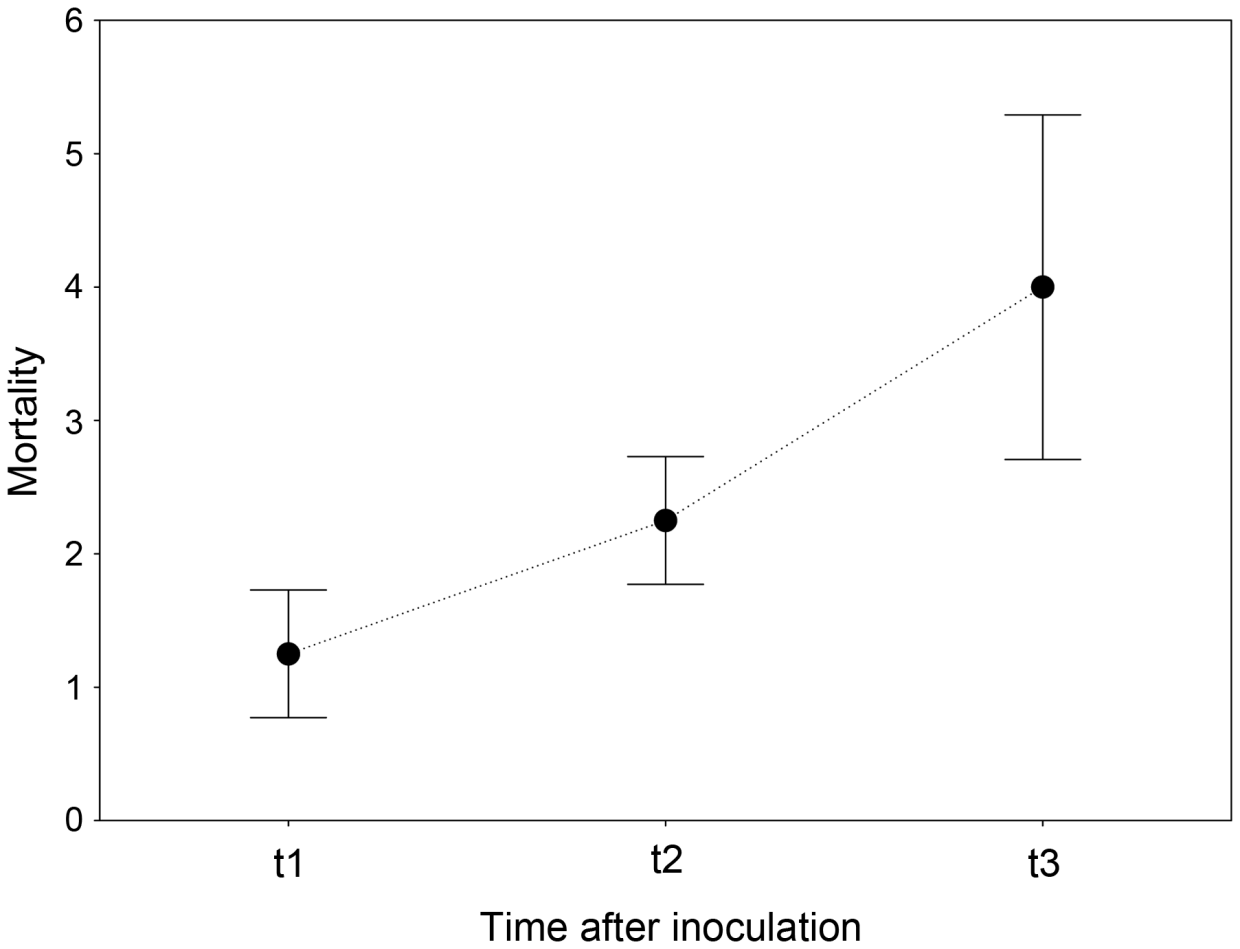
Adult mortality (mean ± s.e.) of *T. lusitanicus* fed on cow dung after exposure to conidia of *M. anisopliae*. Time period: t1 = 4 days, t2 = 8 days and t3 = 12 days.

None of the individuals that were fed acorns (n = 120) died within twelve days of inoculation with the pathogen, while a total of 30 individuals died when a dung diet was used. Of the beetles fed on dung, only time seemed to be marginally significant (*F* = 3.32; *P* = 0.06). Mortality increased eight days after exposure to *M. anisopliae* (45%) compared to the mortality at the beginning of the experiment (12.5%) (Fig. 4).

## Discussion

The present study demonstrated that a shift in the diet of a typical dung beetle from cow dung to a protein-rich diet based on acorns increased resistance towards a generalist pathogen and decreased adult mortality. These results, together with those obtained in a previous study [29], suggest that an apparently simple shift in diet can drastically and potentially enhance the fitness and survival of an insect species.

### Total Protein Content

A diet with low-quality protein has been associated with metabolic traits that induce high mortality, reduced growth, suspended development, and low reproductive success in insects [4], [40], [41], [42], [43]. The total amount of protein in the haemolymph can be used to predict resistance to different pathogens [4], [6], [21]. Although the scientific basis underlying this relation is still mostly unknown, a positive relation between the total protein content in the haemolymph and proPO and PO activity in *T. lusitanicus* suggest that higher protein concentrations in the haemolymph are associated with higher concentrations of proteins involved in the immune response. This can be further deduced from the response observed upon exposure to *M. anisopliae* conidia. A measurement of protein content in the haemolymph may imply the actions of many different metabolic routes. In the case of *T. lusitanicus*, the expansion of the fat body as a consequence of acorn ingestion coincided with significant variations in the levels of several substances important for ecophysiological functions, such as fatty-acids and proteins [29]. This association suggests that fat body quantification is an indirect measurement of immune challenge [44], [45]. This finding is especially true if we consider that the fat body is responsible for the synthesis of the serpin-family inhibitor Necrotic, which is subsequently endocytosed by garland and pericardial athrocytes [46]. Our results corroborated the effect of diet on haemolymph protein content, but they also revealed a positive relation between the total protein content in the haemolymph and proPO and PO activity. However, it should be noted that many proteins in the insect haemolymph are not related to immunity [47]. Therefore, the use of total protein content as a predictive measure of insect resistance to disease should be considered with caution.

### ProPO and PO Activity

Both the proPO and PO levels in the haemolymph of *T. lusitanicus* were dependent on the type of diet. Thus, a diet based on acorns resulted in higher immune responses than a diet based on cow dung. The PO levels were more variable compared to the proPO levels. According to the results, the beetles fed with acorns developed a more effective proPO-PO system than the beetles fed with cow dung. According to the variation observed in the proPO levels, the pathogen exposure likely induced only a small level of proPO to be converted into PO, as occurs in other insects [48], [49], and the observed increased levels of proPO provide fewer benefits compared to increased PO. Moreover, PO activity should be enhanced only when necessary, as observed in the relation of PO activity with conidia exposure time (Figure 2B). For these reasons, we believe that the conditions of pathogen exposure in this study were more likely to induce an immune response by PO than by proPO.

### Pathogen Resistance and Mortality

In our study, the effect of diet on the survival of *T. lusitanicus* upon infection by *M. anisopliae* has been highlighted. Significant increases in PO activity by the beetles fed with acorns suggest a clear immune response to the pathogen. However, the results obtained in the survival analysis of *T. lusitanicus* fed on acorns suggest the existence of synergistic mechanisms that can multiply the positive effects of PO on immunity upon exposure to *M. anisopliae* conidia. Acorns of the *Quercus* species contain large amounts of secondary metabolites, such as phenolic compounds [50], [51], which are considered important for antimicrobial and anti-disease applications [52]. During the innate immune response of invertebrates, active PO catalyses the oxidation of phenolic compounds to quinones and melanin, which are highly toxic to infectious microorganisms [12]. Thus, although more research is needed to examine the possible interactions among different mechanisms of the innate defence system of *T. lusitanicus*, we suggest that a diet based on acorns may be useful for preventing diseases during the autumn-winter period when high energy-demand processes, such as reproduction and hibernation, usually occur [29].

### Conclusions

The present study suggests that increased PO activity in response to an immune challenge reflects resistance to the entomopathogenic fungus *M. anisopliae*. This supposition is supported by the significant increase in the mortality of infected individuals submitted to a dung-based diet and the survivorship of beetles submitted to a acorn-based diet. It is unlikely that a single immune measure will represent the resistance capacity against different diseases [53]. However, the selection of a natural pathogen for *T. lusitanicus*, *M. anisopliae*, suggests that a diet enriched with acorns during the autumn-winter period could be important for preventing diseases during hibernation and increasing the overwintering survival. This increased immune response, as well as the higher cryoprotectant content found in the haemolymph [29] of acorn-fed beetles, were associated features that jointly increased resistance to entomopathogens and cold tolerance. For an overwintering species, such as *T. lusitanicus*, the ability to increase the immune response and cold tolerance may be advantageous, not only because it enables the beetles to survive winter, especially during starvation, which decreases immunity in insects [54], but also by extending their activity during the winter when others species remain inactive and become more susceptible to diseases [29].

Based on these results, it would be interesting to further analyse the benefits of a diet enriched with acorns during the autumn-winter period. The increase in total protein content may not only affect the immune system but also other systems, and it is therefore important to analyse other aspects that may be affected by this change. Moreover, an improved immune system may bring benefits against compounds other than entomopathogens, which may be key to the survival and maintenance of *T. lusitanicus* populations.
